# Skin health of Aboriginal children living in urban communities

**DOI:** 10.1111/ajd.14363

**Published:** 2024-08-28

**Authors:** Bernadette M. Ricciardo, Heather‐Lynn Kessaris, Noel Nannup, Dale Tilbrook, Nadia Rind, Richelle Douglas, Jodie Ingrey, Jacinta Walton, Carol Michie, Brad Farrant, Eloise Delaney, S. Prasad Kumarasinghe, Jonathan R. Carapetis, Asha C. Bowen

**Affiliations:** ^1^ University of Western Australia Crawley Western Australia Australia; ^2^ Wesfarmers Centre of Vaccines and Infectious Diseases Telethon Kids Institute Nedlands Western Australia Australia; ^3^ Perth Children's Hospital Nedlands Western Australia Australia; ^4^ Fiona Stanley Hospital Murdoch Western Australia Australia; ^5^ Telethon Kids Institute Nedlands Western Australia Australia; ^6^ Maalingup Aboriginal Gallery Caversham Western Australia Australia; ^7^ Derbarl Yerrigan Health Service Aboriginal Corporation East Perth Western Australia Australia; ^8^ South West Aboriginal Medical Service Bunbury Western Australia Australia; ^9^ Western Dermatology Nedlands Western Australia Australia

**Keywords:** Aboriginal Community Controlled Health Organisations, Australian Aboriginal and Torres Strait Islander Peoples, child, dermatology, skin, urban

## Abstract

**Background:**

Skin concerns are frequent among urban‐living Aboriginal children, yet specialist dermatology consultations are limited with studies highlighting the need for improved cultural security. Through newly established paediatric dermatology clinics at two urban Aboriginal Community Controlled Health Organisations (ACCHOs), we aimed to describe clinic and patient data, including disease frequencies and associations, to inform dermatology service provision and advocacy.

**Methods:**

A prospective cohort study of Aboriginal children and young people (CYP, 0–18 years) attending Aboriginal Health Practitioner (AHP) co‐ordinated paediatric dermatology clinics at two urban ACCHOs.

**Results:**

Data were collected from 32 clinics over 19 months, with 335 episodes of care and a mean attendance rate of 74%. From 78 new patients, 72 (92%) were recruited into the study, only one of whom had previously received dermatologist assessment. Eczema, tinea or acne accounted for 47% (34/72) of referrals, and 60% of patients received their first appointment within 4 weeks of referral. In 47/72 (65%) consultations, the GP referral and dermatologist diagnosis concurred. The most frequent diagnoses (primary or secondary) at first consultation were atopic dermatitis (26%, 19/72), dermatophyte infections (25%, 18/72), acne (21%, 15/72), bacterial skin infections (18%, 13/72) and post‐inflammatory dyspigmentation (18%, 13/72). Three categories of the 2022 Australasian College of Dermatologists curriculum (infections, eczema/dermatitis, pigmentary disorders) accounted for 59% of all diagnoses.

**Conclusions:**

This study highlights the specialist dermatology needs of urban‐living Aboriginal CYP. ACCHO‐embedded dermatology clinics co‐ordinated by AHPs demonstrated benefits for Aboriginal CYP in accessing care. Opportunities to embed dermatology practice within ACCHOs should be prioritised.

## INTRODUCTION

Skin conditions represent 16–22% of primary care consultations for Aboriginal people in Australia.^1^, ^2^ For urban‐living Aboriginal children and young people (CYP, 0–18 years), at least one dermatological diagnosis is made in more than a quarter of primary care consultations.^3^ Whilst many of these conditions can and should be managed in primary care, there is a need for timely and culturally sensitive specialist care to support general practitioners (GP) treating Aboriginal CYP with more complex dermatological needs.

In our setting of Western Australia (WA), nearly 60% of all Aboriginal CYP reside in urban settings (major cities and inner regional).^4^ The majority of public dermatology outpatient appointments for CYP are available at two tertiary hospitals in Perth. Attendance rates for Aboriginal CYP are lower than their non‐Aboriginal peers.^5^ Previous Australian studies have described specialist dermatology care of Aboriginal patients (Table 1), with two urban studies highlighting the need to improve the cultural security required for Aboriginal people to access tertiary hospital dermatology services.^5^, ^6^

**TABLE 1:** Australian studies describing specialist dermatology care of Aboriginal patients.

Author (year published)	Study design. Data collection period	Healthcare setting (*n*). Geographical location	Aboriginal participants	Mean clinic attendance rate for aboriginal participants	Total number of diagnoses among aboriginal participants made by a dermatologist	Most frequent diagnostic categories among aboriginal participants (*n*)
Heyes et al. (2011)	Retrospective audit. 7 months.	Urban tertiary hospitals (4). Perth, WA.	41 Aboriginal CYP, 63 Aboriginal adults. (39% Aboriginal CYP)	50% for CYP, 48% for adults.	74 (1° and 2° diagnoses from new or review appointments)	Infections^a^ (20) Eczematous conditions (11) Naevi (11) Infestations (5) Psoriasis (4)
Tilakaratne et al. (2016)	Prospective audit. 6 months.	Urban tertiary hospital (1). Adelaide, SA.	2 Aboriginal patients.	–	2 (1° and 2° diagnoses from new or review appointments)	Adnexal diseases (1) Disorders of dermal connective tissue (1)
		Rural tertiary hospital (1), rural hospitals (3) and community clinics (4) (out‐reach service). Darwin, NT. Alice Springs, NT. Nhulunbuy region, NT. Katherine, NT.	41 Aboriginal patients.	–	43 (1° and 2° diagnoses from new or review appointments)^b^	Infections^a^ (22) Skin neoplasms (4) Autoimmune connective tissue disease (4) Eczema/dermatitis (3) Disorders of dermal connective tissue (3)
Haggett et al. (2020)	Retrospective audit. 5 years.	Rural hospitals (5) (out‐reach service). Broome, WA. Derby, WA. Kununurra, WA. Fitzroy Crossing, WA. Halls Creek, WA.	311 Aboriginal patients Median age 39y (IQR: 19.8, 54.3)^c^	–	428 (1° and 2° diagnoses from new or review appointments)^d^	Eczema/dermatitis (80) Autoimmune connective tissue disease (73) Adnexal diseases (42) Pigmentary disorders (32) Benign skin neoplasms (30)
Thomas et al. (2021)	Retrospective cohort study. 12 months.	Rural tertiary hospital (1). Darwin, NT.	61 Aboriginal patients. 24% (26/107) of all episodes of care for Aboriginal patients were for Aboriginal CYP	64% for CYP and adults (combined)	–	–
Williams et al. (2021)	Retrospective audit. 5 years.	Urban ACCHO (1). Melbourne, Victoria.	255 episodes of care (196 new/59 review)^e^ 89% (229/255) for Aboriginal patients 20% (52/255) for CYP^f^	74% for CYP and adults (combined)	163^g^ (1° diagnoses from new appointments)	1 Eczema/dermatitis (28) 2 Premalignant and malignant neoplasms (27) 3 Benign skin neoplasms (20) 4 Infections^b^ (14) 1 Adnexal diseases (10)
Ricciardo et al. (current study) (2024)	Prospective cohort study. 19 months.	Urban ACCHO (2). Perth, WA. Bunbury, WA.	72 Aboriginal CYP (100% Aboriginal CYP)	74% for CYP	223 (1° and 2° diagnoses from new & review appointments	Infections^a^ (59) Eczema/dermatitis (40) Pigmentary disorders (33) Appendageal diseases^h^ (16) Genodermatoses (14)

Abbreviations: 1°, primary; 2°, secondary; ACCHO, Aboriginal Community Controlled Health Organisation; CYP, children and young people (0–18 years); NT, Northern Territory; SA, South Australia; WA, Western Australia.

^a^ Fungal infection most frequently reported.

^b^ Due to time limitations or patient preference many patients did not undergo full skin checks, potentially limiting number of secondary diagnoses.

^c^ Unclear what number/percentage were Aboriginal CYP.

^d^ Excluded ‘Full Skin Examination Normal’ (17) and ‘Uncertain Pathology’ (1).

^e^ Includes those episodes of care where patients did not attend.

^f^ Unclear what number/percentage were Aboriginal CYP.

^g^ Excluded ‘Full Skin Examination Normal’ (33).

^h^ In the 2022 version of the ACD Dermatology Training Programme curriculum, adnexal diseases were renamed as appendageal diseases.

Forming one component of the Koolungar (*children*) Moorditj (*strong*) Healthy Skin (KMHS) project, we aimed to describe clinic and patient data, including disease frequencies and associations, for urban‐living Aboriginal CYP attending newly established ACCHO‐embedded dermatology clinics.^3^, ^7^, ^8^, ^9^ The results will be used to inform dermatology service provision and advocacy.

## METHODS

This project aligns with the Telethon Kids Institute's Guidelines for the Conduct of Aboriginal Health Research.^10^ We have reported according to the Strengthening the Reporting of Observational Studies in Epidemiology (STROBE) guidelines (Appendix S1) and the CONSoliDated critERia for strengthening the reporting of health research involving Indigenous Peoples (CONSIDER) statement (Appendix S2).

### Setting

The KMHS project is the first Aboriginal Elder co‐designed Australian study to comprehensively describe skin health and disease in urban‐living Aboriginal CYP.^8^ Having identified a knowledge gap for skin health in urban‐living Aboriginal CYP, broad consultation with Noongar Elders (Traditional Custodians of south‐western WA) was sought to understand the needs and preferences of community. From here, two Elder Researchers joined the team as investigators, providing Aboriginal leadership and cultural governance to guide all project elements. Through genuine co‐design, the project reflects the perspectives and priorities of the community, upheld by the Elder‐developed guiding principles of respect, reciprocity, capacity building and community involvement.

During co‐design, Elder Researchers prioritised investigating the connection between healthy skin and healthy housing, with the aim of using these results to strengthen advocacy for housing inequities. Elder researchers also considered a research‐service methodology to be integral to study design, ensuring study participants and CYP in the wider community benefit from timely specialist treatment. This led to collaboration with and the establishment of monthly paediatric dermatology clinics at two urban ACCHOs: Derbarl Yerrigan Health Service (Derbarl) on *Whadjuk Noongar* (Perth) *Boodjar* (land/place) and the South West Aboriginal Medical Service (SWAMS) on *Wardandi Noongar* (Bunbury) *Boodjar*.

Taken together, Derbarl (Derbarl Yerrigan Health Service Business Information Unit, unpubl data) and SWAMS (South West Aboriginal Medical Service Business Information Unit, unpubl data) provide integrated primary health care services to approximately 35% of all Aboriginal CYP within their catchments.^11^ They utilise an Aboriginal Health Practitioner (AHP)‐led model of care, supported by visiting non‐GP specialists. AHPs are registered with the Australian Health Practitioner Regulation Agency to provide high‐quality, culturally safe healthcare to Aboriginal people and communities. Within ACCHOs, AHPs facilitate age‐appropriate assessments prior to GP consultation and support GP and visiting specialist clinics. In the KMHS project, funding was obtained for ACCHO‐embedded AHPs to join the clinical and research team; they were trained in Good Clinical Practice with the opportunity provided to complete the Dermatology Australasia Aboriginal Health Workers Course. In addition to co‐ordinating and assisting in the clinic, the AHP role included confirming appointments and arranging transport assistance where needed.

### Study design

A prospective cohort study of Aboriginal CYP attending newly established paediatric dermatology clinics at two urban ACCHOs.

### Study population

Urban‐living Aboriginal CYP (0‐18y) attending face‐to‐face new patient consultation were eligible for recruitment. *Urban‐living* included residents of the Australian Government Department of Health and Aged Care 2021 Modified Monash category one (MM1; metropolitan areas) or two (MM2; regional centres).

### Recruitment

Data collection periods were 01.09.2021–01.04.2023 for Derbarl (19 months) and 01.04.2022–01.04.2023 for SWAMS (12 months). At the start of the consultation, the patient and caregiver were invited to participate in the study; a participant information sheet was provided, and written informed consent obtained by the AHP or dermatologist.

### Data collection

#### Clinic data

Data were collected at completion of each clinic and stored in a REDCap database, including total number of episodes of care, clinic outcome (attended, non‐attended and rescheduled), consultation type (new, review) and delivery mode (face‐to‐face, telehealth).

#### Participant data

A case report form (CRF) was used to collect demographic and prospective data from patient history, skin examination, investigations, treatment and disposition. Additional information was gathered to comprehensively describe skin health and align with Elder Researcher priorities: traditional bush medicine for skin health, sunburn and sunscreen use, skin infection risk factors and complications, atopy and nutritional deficiency, household structure and health hardware. Completed CRFs were added to the medical record and de‐identified information captured in REDCap, including follow‐up visits and clinical photos.

### Statistical analysis

Data were analysed using R version 4.1.2. Summary statistics were calculated. The Eczema Area and Severity Index (EASI) was used to assess atopic dermatitis (AD), with cut‐off points of <7, 7–21 and >21 indicating mild, moderate and severe disease.^12^ Skin of colour (SOC) was defined as Fitzpatrick Skin Phototype (FSP) IV‐VI.^13^ The total number and breakdown of dermatological diagnoses (primary or secondary) at initial and follow‐up consultations were calculated. Diagnoses were classified into one of the 42 Specialised Content Topic Areas (SCTA) of the 2022 Australasian College of Dermatologists (ACD) training programme curriculum to facilitate comparison with other Australian studies.^5^, ^6^, ^14^, ^15^, ^16^ The five most frequent dermatological diagnoses (primary or secondary) at initial consultation were calculated as a proportion of participants. For these diagnoses, descriptive statistics were calculated with odds ratios (OR) and 95% confidence intervals (CI) (±*p*‐values where CI were borderline) to investigate frequency variations by demographics, antenatal/medical history, skin care routine and household structure/health hardware.

### Dissemination

A thank you letter with summarised results, along with the KMHS project newsletter detailing wider project news, were shared with participating families. The findings have been co‐presented at international, local and community meetings.

### Ethics

Ethics approval was granted by the WA Aboriginal Health Ethics Committee (WAAHEC) and the University of WA.

## RESULTS

### Clinic details

**FIGURE 1:**
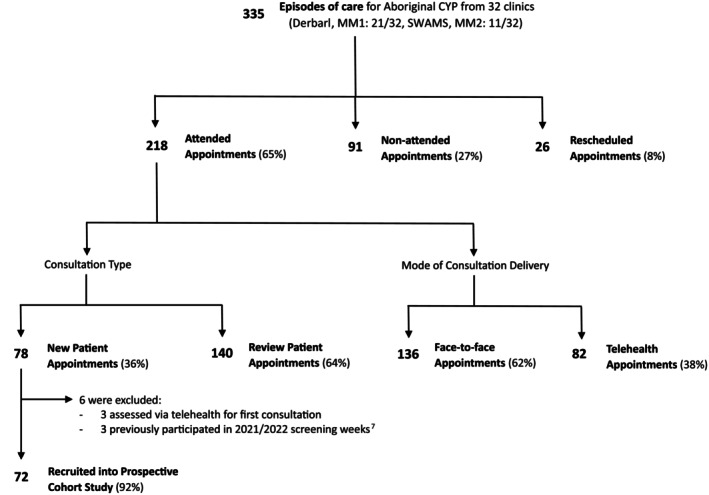
Consort flow diagram. CYP, Children and Young People (0–18 years), MM1, Modified Monash 1 (metropolitan areas); MM2, Modified Monash 2 (regional centres), Derbarl = Derbarl Yerrigan Health Service, SWAMS = South West Aboriginal Medical Service.

Data were collected from 32 clinics over 19 months, with 335 episodes of care and a mean attendance rate of 74% (standard deviation: 14.4) (Figure 1). Rescheduled appointments accounted for 8% (26/335) of episodes of care. Of the 218 attended appointments, there were 78 episodes of care for new patients (36%) and 140 for reviews (64%), including follow‐ups from the 2021/22 community skin screening weeks.^8^

### Participant demographics

From 78 new patients, 72 urban‐living Aboriginal CYP were recruited (92%); median age 6 years (interquartile range [IQR]: 3–13), 53% (38/72) female and 68% (49/72) with SOC.

### Referral details

Most participants received their first appointment within 4 weeks of referral (43/72, 60%), including 28% (20/72) seen on the day of referral. There were 83 clinical indications documented in the 72 referrals: one indication in 86% (62/72), two in 13% (9/72) and three in 1% (1/72). Referrals were for skin rash (72%), hair concern (15%), skin lesion (8%) and nail concern (4%), with eczema (18%), tinea (18%) and acne (11%) the most frequent indications. Prior to referral, baseline investigations were ordered and treatments trialled in 24% (17/72) and 68% (49/72), respectively. 1/72 (1%) participants had previous dermatologist assessment.

### Dermatological diagnoses

In 47/72 (65%) consultations, the GP referral and dermatologist diagnosis concurred. In total, 205 dermatological diagnoses were made in the 72 CYP at their first consultation, an average of 2.8 diagnoses/CYP, with dermatological diagnoses increasing by 250% (205 vs. 83) following dermatologist assessment (Table 2). Fourteen percent (10/72) of participants had one diagnosis, 24% (17/72) had two, 32% (25/72) had three, 24% (15/72) had four, 6% (4/72) had five and 1% (1/72) had six. A further 18 diagnoses were made in 13/51 (25%) participants who attended follow‐up. Applying the 2022 ACD Curriculum to the 223 total dermatological diagnoses included 16/42 (38%) SCTA (Appendix S3): infections, eczema/dermatitis and pigmentary disorders accounted for 59% (132/223) (Table 3).

**TABLE 2:** Dermatological diagnoses at initial and follow‐up consultations.

Dermatological diagnoses	Initial consultation			Follow‐up consultation diagnoses	Total
	Primary diagnosis^a^	Secondary diagnoses^b^	Combined primary and secondary diagnoses		
Atopic dermatitis	16 (22%)	3 (2%)	19 (9%)	1 (6%)	20 (9%)
Acne	7 (10%)	8 (6%)	15 (7%)	1 (6%)	16 (7%)
Post‐inflammatory dyspigmentation^c^	0	13 (10%)	13 (6%)	1 (6%)	14 (6%)
Bacterial skin infection^d^	4 (6%)	9 (7%)	13 (6%)	1 (6%)	14 (6%)
Tinea capitis	8 (11%)	5 (4%)	13 (6%)	0	13 (6%)
Tinea corporis	3 (4%)	8 (6%)	11 (5%)	1 (6%)	12 (5%)
Seborrhoeic dermatitis	1 (1%)	10 (9%)	11 (5%)	0	11 (5%)
Keratosis pilaris	4 (6%)	6 (5%)	10 (5%)	1 (6%)	11 (5%)
Viral verruca	3 (4%)	6 (5%)	9 (4%)	1 (6%)	10 (4%)
Pigmentary mosaicism^e^	0	9 (7%)	9 (4%)	0	9 (4%)
Dermatitis – ‘other’^f^	2 (3%)	3 (2%)	5 (2%)	3 (17%)	8 (4%)
Scarring – hypertrophic, keloid	3 (4%)	5 (4%)	8 (4%)	0	8 (4%)
Pediculosis capitis	0	5 (4%)	5 (2%)	2 (11%)	7 (3%)
Molluscum contagiosum	3 (4%)	3 (2%)	6 (3%)	0	6 (3%)
Acanthosis nigricans ± acrochordons	0	6 (5%)	6 (3%)	0	6 (3%)
Nail disorder (non‐infectious)^g^	3 (4%)	3 (2%)	6 (3%)	0	6 (3%)
Pityriasis alba	1 (1%)	3 (2%)	4 (2%)	1 (6%)	5 (2%)
Infantile haemangioma	2 (3%)	2 (2%)	4 (2%)	0	4 (2%)
Scarring – atrophic, striae	0	4 (3%)	4 (2%)	0	4 (2%)
Ichthyosis (inherited non‐syndromic form)	2 (3%)	1 (1%)	3 (1%)	0	3 (1%)
Malassezia (pityrosporum) folliculitis	0	3 (2%)	3 (1%)	0	3 (1%)
Naevus simplex	0	3 (2%)	3 (1%)	0	3 (1%)
Hair disorder (non‐infectious)^h^	0	3 (2%)	3 (1%)	0	3 (1%)
Scabies	0	1 (1%)	1 (<1%)	2 (11%)	3 (1%)
Sunburn	0	2 (2%)	2 (1%)	1 (6%)	3 (1%)
Melanocytic naevus – benign	2 (3%)	0	2 (1%)	0	2 (1%)
Pityriasis versicolor	2 (3%)	0	2 (1%)	0	2 (1%)
Vitiligo	1 (1%)	1 (1%)	2 (1%)	0	2 (1%)
Urticaria – chronic, intermittent episodic and acute	2 (3%)	0	2 (1%)	0	2 (1%)
Congenital abnormality^i^	0	2 (2%)	2 (1%)	0	2 (1%)
Fibrous papule(s) of the nose^j^	0	1 (1%)	1 (<1%)	1 (6%)	2 (1%)
Tinea unguium (onychomycosis)	1 (1%)	0	1 (<1%)	0	1 (<1%)
Papular urticaria	1 (1%)	0	1 (<1%)	0	1 (<1%)
Idiopathic eruptive macular hypermelanosis	1 (1%)	0	1 (<1%)	0	1 (<1%)
Hyperkeratotic flexural erythema	0	1 (1%)	1 (<1%)	0	1 (<1%)
Primary idiopathic palmoplantar hyperhidrosis	0	1 (1%)	1 (<1%)	0	1 (<1%)
Spider naevi/telangiectasia	0	1 (1%)	1 (<1%)	0	1 (<1%)
Epidermal cyst	0	1 (1%)	1 (<1%)	0	1 (<1%)
Callus – frictional	0	1 (1%)	1 (<1%)	0	1 (<1%)
Confluent and reticulated papillomatosis	0	0	0	1 (6%)	1 (<1%)
Total	72	133	205	18	223

^a^ Each participant can only have one primary diagnosis.

^b^ Each participant can have multiple secondary diagnoses.

^c^ Post‐inflammatory dyspigmentation includes post‐inflammatory hyperpigmentation and post‐inflammatory hypopigmentation.

^d^ Bacterial skin infection refers to primary impetigo and secondary bacterial infection of underlying dermatosis.

^e^ Pigmentary mosaicism includes café‐au‐lait macules.

^f^ Dermatitis ‘other’ includes irritant contact dermatitis (diaper dermatitis, intertrigo), phyto‐photo‐dermatitis, lichen simplex chronicus and juvenile plantar dermatosis.

^g^ Nail disorder (non‐infectious) includes congenital malalignment of the great toenails, traumatic onychodystrophy, onychomadesis and paronychia.

^h^ Hair disorder (non‐infectious) includes generalised hypertrichosis, hirsutism and loose anagen hair syndrome.

^i^ Congenital abnormality includes aplasia cutis and pre‐auricular sinus.

^j^ Fibrous papule(s) of the nose include angiofibroma and angiofibroma‐like nasal papules associated with acne.

**TABLE 3:** Dermatological diagnoses at initial and follow‐up consultations applied to the 2022 Australasian College of Dermatologists curriculum.

ACD specialised content topic area	Diagnoses made at initial consultation	Diagnoses made at follow‐up consultations	Total
1. Infections	56 (27%)	3 (2%)	59 (26%)
2. Eczema/dermatitis	36 (18%)	4 (2%)	40 (18%)
3. Pigmentary disorders	31 (15%)	3 (2%)	33 (15%)
4. Appendageal diseases	15 (7%)	1 (1%)	16 (7%)
5. Genodermatoses	13 (6%)	1 (1%)	14 (6%)
6. Disorders of dermal connective tissue	12 (6%)	0	12 (5%)
7. Infestations, bites and stings	7 (3%)	4 (2%)	11 (5%)
8. Developmental disorders/hamartoma	9 (4%)	0	10 (4%)
9. Skin signs in patients with systemic disease	6 (3%)	0	6 (3%)
10. Disorders of nails	6 (3%)	0	6 (3%)
11. Benign skin neoplasms	4 (2%)	1 (1%)	5 (2%)
12. Disorders due to physical agents	3 (1%)	1 (1%)	4 (2%)
13. Disorders of hair	3 (1%)	0	3 (1%)
14. Urticaria	2 (1%)	0	2 (1%)
15. Vascular system disorders	1 (0.05%)	0	1 (0.5%)
16. Disorders of eccrine or apocrine glands	1 (0.05%)	0	1 (0.5%)
Total	205	18	223

**FIGURE 2:**
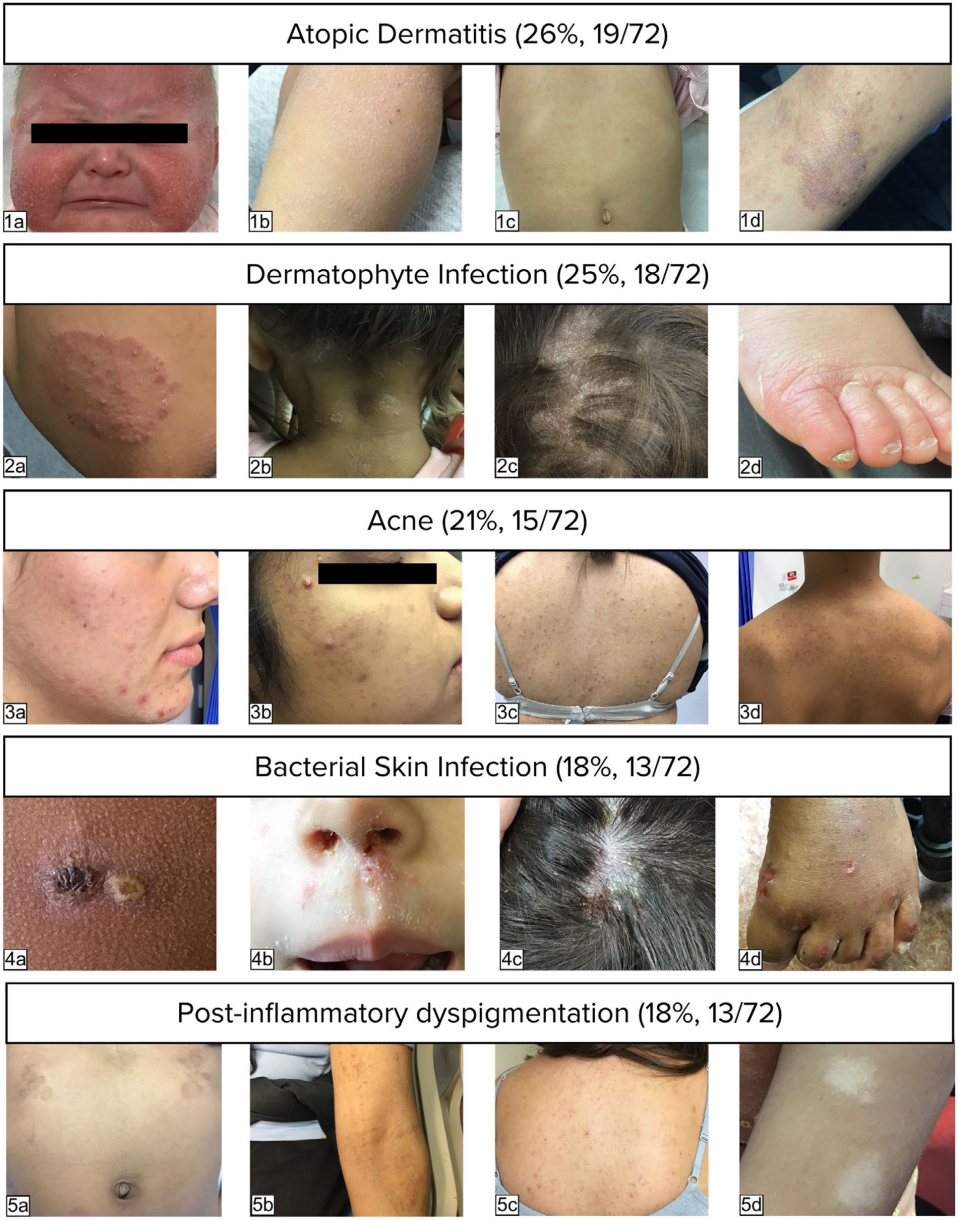
Most frequent diagnoses (primary/secondary) at initial consultation as a proportion of participant number – (1) Atopic dermatitis (AD): (a) generalised AD in an infant, (b) generalised AD in a toddler, (c) papular AD affecting the torso of a toddler and (d) lichenified flexural AD in a teenager. (2) Dermatophyte infections: (a) tinea corporis of the elbow, (b) tinea corporis of the neck and tinea capitis, (c) tinea capitis and (d) tinea pedis and onychomycosis. (3) Acne: (a/b) comedonal and papulopustular facial acne with post‐inflammatory hyperpigmentation and (c/d) comedonal and papulopustular torso acne with post‐inflammatory hyperpigmentation. (4) Bacterial skin infections (BSI): (a) primary impetigo, (b) secondary BSI of AD, (c) secondary BSI of tinea capitis and pediculosis capitis and (d) secondary BSI of scabies. (5) Post‐inflammatory dyspigmentation: (a) hyperpigmentation secondary to tinea corporis, (b) hyperpigmentation secondary to AD, (c) hyperpigmentation secondary to acne and (d) hypopigmentation secondary to AD.

The five most frequent diagnoses at initial consultation (Figure 2) are described below:

#### Atopic dermatitis

AD was diagnosed in 19/72 (26%) participants at initial consultation; median age 3 years (IQR: 1–7) and 53% (10/19) female. Forty‐two percent (8/19) were using a soap‐free wash and regular emollient, and 26% (5/19) a prescription topical corticosteroid. Allergic rhinitis, iron deficiency and asthma were reported in 21% (4/19), 16% (3/19) and 11% (2/19), respectively. No participant with AD recalled a diagnosis of vitamin D deficiency (0/19), including 58% (11/19) with SOC. The presence of household furry pets was the only predictive factor, present in 68% (13/19; OR 3.42, 95% CI 1.11–10.54). AD was moderate to severe in 42% (8/19), with secondary BSI in 21% (4/19) and post‐inflammatory dyspigmentation (PID) in 26% (5/19). Thirty‐two percent (6/19) were discharged to GP after initial appointment, with 42% (8/19) requiring one review, 16% (3/19) two to four and 11% (2/19) still receiving care at study closure.

#### Dermatophyte infections

Dermatophyte infections were diagnosed in 18/72 (25%) participants from 14 households at initial consultation; median age 5.5 years (IQR: 3–9) and 39% (7/18) female. Bathroom plumbing/maintenance issues (OR 4.67, 95% CI 1.0–23.26, *p*‐value 0.046) and bed‐sharing sometimes/always (OR 5.3, 95% CI 1.65–16.37), along with a past history of BSI (OR 6.91, 95% CI 2.15–22.28) and scabies (OR 4.9, 95% CI 1.28–18.79), predicted dermatophyte infection. Scalp tinea was diagnosed in 7/18 (39%), scalp plus skin in 6/18 (33%), skin alone in 4/18 (22%) and skin plus nails in 1/18 (6%). Of the 13 participants with tinea capitis, 69% (9/13) described months to years of scalp scaling, 23% (3/13) were an incidental finding and 8% (1/13) presented with a kerion. Secondary/concurrent BSI was present in 5/18 (28%), while PID affected 2/11 (18%) with tinea corporis. 100% (10/10) of collected hair pluck specimens for tinea capitis cultured *Trichophyton tonsurans. T. rubrum* was cultured from the one participant with onychomycosis.

Oral terbinafine was prescribed as first‐line treatment for the 14 participants with onychomycosis (1/18) or tinea capitis (13/18), in combination with ketoconazole 2% shampoo in the latter. If required, parents were advised to crush the tablet and mix with a teaspoon of yoghurt, Nutella® or similar. 50% (7/14) did not tolerate terbinafine due to inability to swallow tablets, grainy texture when crushed, or bitter taste. Where feasible, compounded griseofulvin liquid was prescribed as second‐line therapy (43%, 3/7); however, poor compliance due to unpleasant taste and duration was reported. All participants with dermatophyte infection received follow‐up: 39% (7/18) requiring one review, 50% (9/18) two to four and 11% (2/18) still receiving care at study closure.

#### Acne

Acne was diagnosed in 15/28 (54%) 10 to 18‐year‐old participants at initial consultation; median age 14 years (IQR: 12–16) and 67% (10/15) female. Iron deficiency was reported in 7/10 (70%) affected females. 6/15 (40%) affected participants had comedonal acne and 9/15 (60%) had combination comedonal and inflammatory acne. PID was present in 6/15 (40%). Systemic acne therapy was prescribed in 7/15 (47%), with 4 receiving isotretinoin. Discharge to GP after initial consultation occurred in 6/15 (40%), with 4/15 (27%) still receiving care at study closure.

#### Bacterial skin infections

BSI were diagnosed in 13/72 (18%) participants at initial consultation; median age 3 years (IQR: 2–6.5) and 23% (3/13) female (OR 0.21, 95% CI 0.05–0.83). Bedsharing sometimes/always was the only predictive factor, present in 62% (8/13; OR 3.37, 95% CI 0.97–11.69, *p*‐value 0.049). Primary impetigo was diagnosed in 5/13 (38%); with BSI secondary to underlying dermatosis in 8/13 (62%): eczema/dermatitis (3/8), dermatophyte infection (2/8), nail disorder (2/8) and molluscum contagiosum (1/8). Of 11 skin swabs taken, *Staphylococcus aureus* was cultured in five (45%), *S. aureus* and *Streptococcus pyogenes* in four (36%) and *S. pyogenes* in one (9%). BSI were treated with oral antibiotics (co‐trimoxazole, cephalexin or flucloxacillin) in 12/13 (92%) and topical mupirocin in 1/13 (8%). Anterior nares swab cultured *S. aureus* in 50% (3/6) of participants with recurrent BSI, and staphylococcal decolonisation was prescribed.

#### Post‐inflammatory dyspigmentation

PID was diagnosed in 13/72 (18%) participants at initial consultation; median age 10.5 years (IQR: 2.5–15) and 46% (6/13) female. One hundred percent (13/13) of participants with PID had FSP III‐V: 6/13 (46%) reported past sunburn, 9/13 (69%) described never using sunscreen and no history of vitamin D deficiency was recalled (0/13). PID was secondary to acne in 46% (6/13) where it presented with hyperpigmentation in all cases (6/6), eczema/dermatitis in 38% (5/13) with hyperpigmentation in 2/5 cases and hypopigmentation in 3/5 cases and tinea corporis in 15% (2/13) with hyperpigmentation in all cases (2/2).

### Disposition

Following initial consultation, 14/72 (19%) participants were discharged to their GP, 54/72 (75%) required follow‐up in the clinic and 4/72 (6%) were referred to tertiary hospitals for surgery or laser. Of the 54 scheduled reviews, the majority (21/54, 39%) required only one review, 14/54 (26%) two to four reviews, 16/54 (30%) still receiving care at study closure and 3/54 (6%) lost to follow‐up.

## DISCUSSION

This study focuses on the specialist dermatology needs of urban‐living Aboriginal CYP. Our results support embedding dermatology clinics alongside primary care in an ACCHO setting to facilitate timely access and optimise attendance. The ongoing need for dermatology services is highlighted by the 250% increase in dermatological diagnoses at first consultation enabling knowledge and treatment in CYP of whom only 1% had ever seen dermatology, and with one third of patients receiving their first accurate diagnosis and treatment plan. Three categories of the 2022 ACD curriculum (infections, eczema/dermatitis, pigmentary disorders) accounted for nearly 60% of all diagnoses, highlighting areas where dermatology trainees require expertise in clinical care and health advocacy.

To achieve better health care for Aboriginal CYP, tertiary hospitals should continue in their responsibility and efforts to provide inclusive services, including building relations with local ACCHOs to embed specialist care. Previous studies have highlighted barriers for urban‐living Aboriginal patients attending hospital outpatient appointments, along with facilitators including varied appointment reminder formats, flexible appointments, transport support, food in waiting rooms, outreach services, access to Aboriginal support workers, improving communication and relationships with Aboriginal people, cultural awareness training for staff and provision of culturally appropriate spaces, all of which are provided within the ACCHO setting.^17^, ^18^, ^19^ Community engagement with a novel clinical service and the favourable attendance rates seen in our study, replicated in other ACCHO‐embedded speciality clinics, reflect the culturally safe environment and AHP‐led model of care.^6^, ^20^ AHPs play an integral role in health service delivery within ACCHOs, positively impacting health service utilisation and patient outcomes.^21^ In addition, prioritising employment and retention of Aboriginal staff within ACCHOs supports the provision of culturally appropriate care in the services that they and their non‐Aboriginal colleagues deliver.^22^, ^23^

Underutilisation of tertiary hospital dermatology services by Aboriginal patients has been reported.^5^ Of the 72 patients recruited into our study, only one previously had access to dermatology. Following the first consultation, 122 additional diagnoses were made surplus to the 83 indications on GP referrals; and 35% of participants received an accurate dermatologist diagnosis, allowing for education on their condition and the formulation of an effective management plan. Previous studies have identified GP limitations in accurately diagnosing skin disease, with rates varying from 26% to 57%; the highest for conditions such as acne (100%), warts (100%) and molluscum contagiosum (100%), and lower for *S. aureus* infection (67%), dermatitis (64%) and fungal infection (25%).^24^ While this highlights the need for further dermatology education at a primary care level, it also affirms the ongoing need for specialist dermatologist advice.

A significant burden of BSI and AD exists in urban‐living Indigenous children in high‐income countries.^9^ In our study, infections and eczema/dermatitis were the most frequent diagnostic categories; accounting for 26% and 18% of all diagnoses, respectively. This concurs with a retrospective audit of Aboriginal patients (39% CYP patients) attending hospital dermatology clinics in Perth, WA, where skin infections and eczematous conditions were most frequent and accounted for 27% (20/74) and 15% (11/74) of primary/secondary diagnoses, respectively.^5^ Eczema/dermatitis was the most frequent diagnostic category for urban‐living Aboriginal patients (20% CYP episodes of care) assessed in Melbourne, Victoria, with the smaller proportion of CYP perhaps explaining why infections ranked fourth.^6^

The high burden of skin infections well described in remote‐living Aboriginal CYP, and more recently in urban‐living Aboriginal CYP, can be attributed to the social determinants of health that are the consequence of colonisation, dispossession and ongoing systemic racism.^3^, ^8^, ^25^, ^26^ For many affected CYP and their families, this includes poor living conditions and lack of access to well‐maintained health hardware, resulting in ongoing transmission of fungal, bacterial and parasitic skin infections.^27^ The clustering of skin infections and predictive factors identified in this study add to a growing body of evidence that suggests there are urban‐living Aboriginal CYP living in housing conditions that may be impacting on their health, supporting the need for investment in housing and housing maintenance to improve health for urban‐living Aboriginal CYP.^8^

Pigmentary disorders are common concerns in patients with SOC consulting dermatology.^28^ In our study, where 68% of participants had SOC, pigmentary disorders were the third most common diagnostic category. PID was the main contributor (42%); with all affected CYP displaying FSP III‐V, consistent with published literature, and most frequently secondary to acne.^29^ While PID is well described in many SOC populations where it is associated with emotional distress leading to reduced quality of life, including in young people with acne, PID is not well described in Australian Aboriginal patients.^30^, ^31^ In fact, only one of four Australian studies reporting diagnostic frequencies in Aboriginal patients references PID specifically (Table 1); a disorder we suspect is underdiagnosed, undertreated and causing unknown psychosocial impact.^5^ Further, ultraviolet exposure is known to be an external trigger for PID and our study revealed high rates of sunburn in participants with PID (46%) and low rates of sunscreen use (31%).^32^ This is also described in other SOC populations, with photoprotection shown to improve pigmentary concerns, including PID.^13^ Education on photoprotection to minimise PID is imperative for Aboriginal CYP with SOC, in addition to clinician education on PID diagnosis and management.^13^

To improve dermatological health outcomes for Aboriginal CYP, and other paediatric populations with SOC, expertise in diagnosis and management of skin infections, eczema/dermatitis and pigmentary disorders should be prioritised for dermatology trainees and can be enhanced through their inclusion in ACCHO‐embedded dermatology clinics. A national survey of Australian dermatologists revealed >80% would have liked more education and training on SOC.^33^ Increased clinical exposure during dermatology training has been shown to improve confidence and knowledge in the diagnosis and management of dermatological disorders in SOC.^34^, ^35^ Trainees also develop a better understanding of the health disparities facing Aboriginal patients, resulting from inequities in the social determinants of health.^26^ In the ACCHO setting, trainees learn practical knowledge of government schemes for Aboriginal patients to improve treatment outcomes, including the Australian Government Department of Health and Aged Care's *Closing the Gap PBS Co‐payment Program* and *Listings on the PBS for Aboriginal and Torres Strait Islander people*; as well as ACCHO‐specific formularies and practices. Trainees can access patient educational resources and treatment guidelines specifically developed and evaluated for use in Aboriginal CYP (telethonkids.org.au).^7^, ^36^

Important patient advocacy skills are learnt in the ACCHO setting. In our study, clinician observation and parent feedback suggested provision of non‐prescription products for AD management, the most frequent diagnosis in our cohort, helped optimise disease control. AD‐related out‐of‐pocket costs for non‐prescription items pose a substantial burden on families, being higher than other chronic diseases.^37^ Lobbying the government for non‐prescription AD items to be subsidised for financially vulnerable community members is an important advocacy role of the ACD. Similarly, in tinea capitis, the fifth most frequent diagnosis in our cohort, we encountered barriers to treatment with currently available systemic anti‐fungal therapy, some of which have been described.^38^ This clinical problem was brainstormed with the Elder Researchers and wider research team, leading to a successful funding application to explore alternative formulations to optimise palatability for children.

There were limitations to our study. The 19‐month study period coincided with the COVID‐19 pandemic; hence, clinic data may not be reflective of non‐COVID periods. Further, clinic data may not reflect established dermatology clinics, where time to first consult may be longer and attendance rates improved with increasing community awareness and confidence in cultural safety. Although this is the largest study of urban‐living Aboriginal CYP attending dermatology, the numbers are modest. The diagnostic frequencies may not be generalisable to other clinical sites and geographical locations, and disease association results are limited by small sample sizes. Univariate analysis compared frequencies of categorical variables between outcome variables, with the inherent risk of confounding. Direct comparison with other Australian studies (Table 1) is challenged by differences in study design, ACD curriculum revisions and diagnostic categories utilised. Our prospective study design, and aim to provide a comprehensive description of skin disease are reflected in the higher frequency of diagnoses reported.

To achieve meaningful improvements in health outcomes for Aboriginal children, we need to, as health practitioners and systems, change our models of care to meet the needs of patients and their families. In this study, ACCHO‐embedded dermatology clinics co‐ordinated by AHPs have led to engagement with specialist care and improved clinical outcomes in skin health for urban‐living Aboriginal CYP. Prioritising Aboriginal health in Aboriginal hands, opportunities for collaboration between ACCHOs and tertiary hospitals or external funding agencies to embed specialist dermatology practice should be sought.

## FUNDING INFORMATION

The Koolungar Moorditj Healthy Skin project is funded by: Wesfarmers Centre of Vaccines and Infectious Diseases (WCVID) Seed Funding and Capacity Building Grants; Channel 7 Telethon Trust Grant and Western Australian Future Health Research & Innovation Fund. With kindly donated products from Cancer Council WA, City of Fremantle, City of Stirling, South West Sports Centre, Maalingup Aboriginal Gallery, Priceline Pharmacy, LaRoche Posay and Ego Pharmaceuticals. BR is the recipient of an Australian Government Research Training Program Fees Offset and WCVID Top‐up Scholarship. The Australian National Health and Medical Research Council provides PhD scholarship funding for BR (GNT2014208) and Investigator Awards for AB (GNT1175509) and JC (GNT1173874).

## CONFLICT OF INTEREST STATEMENT

The authors declare that they have no competing interests.

## ETHICS APPROVAL

Ethics approval was granted by the Western Australian Aboriginal Health Ethics Committee (WAAHEC) [HREC Ref No. 1059] and the University of Western Australia [File Reference – 2021/ET000536].

## PATIENT CONSENT

Written consent for publication obtained for all participants and is available upon request.

## Supporting information


Appendix S1:

Appendix S2:

Appendix S3:

## Data Availability

The datasets generated and/or analysed during the current study are not publicly available due to Indigenous data sovereignty. Requests for the datasets can be made to the corresponding author for consideration by the research team, including Elder Researchers, and ACCHO boards.
