# Supplementary material for: Skin health of Aboriginal children living in urban communities
Source: Australas J Dermatol. 2024 Aug 28;65(8):e224–37. doi: 10.1111/ajd.14363 (PMC11629136; doi:10.1111/ajd.14363)
Supplement: Supplementary file 3 — Appendix S3: [file AJD-65-e224-s003.docx]

##### **Supplementary Material 3** – Specialised Content Topic Areas of the 2022 Australasian College of Dermatologists curriculum

| **ACD Specialised Content Topic Area** | |
| --- | --- |
| **Represented in this Cohort** | **Not represented in this Cohort** |
| 1. Infections ^1^ | 1. Emergency dermatology |
| 1. Eczema / dermatitis ^2^ | 1. Psoriasis |
| 1. Pigmentary disorders ^3^ | 1. Papulosquamous disorders |
| 1. Appendageal diseases | 1. Exanthems |
| 1. Genodermatoses ^4^ | 1. Erythroderma |
| 1. Disorders of dermal connective tissue ^5^ | 1. Erythemas |
| 1. Developmental disorders / hamartoma | 1. Rosecea, periorificial dermatitis and related disorders |
| 1. Infestations, bites, stings | 1. Autoimmune Connective Tissue disease / rheumatologic dermatology |
| 1. Disorders of nails (non-infectious) | 1. Autoinflammatory syndromes |
| 1. Skin signs in patients with systemic disease | 1. Vesiculobullous diseases |
| 1. Benign skin neoplasms ^6^ | 1. Drug reactions |
| 1. Disorders due to physical agents ^7^ | 1. Vasculitis and purpuras |
| 1. Disorders of hair (non-infectious) | 1. Lymphatic system disorders |
| 1. Urticaria | 1. Premalignant and malignant neoplasms |
| 1. Vascular system disorders | 1. Lymphoproliferative and myeloproliferative disorders |
| 1. Disorders of eccrine or apocrine glands | 1. Non-infectious neutrophilic dermatoses |
|  | 1. Eosinophilic dermatoses |
|  | 1. Histiocytosis |
|  | 1. Mastocytosis syndromes |
|  | 1. Disorders of macrophages (non-infectious granulomas) |
|  | 1. Oral diseases |
|  | 1. Anogenital diseases |
|  | 1. Disorders of subcutaneous tissue |
|  | 1. Skin signs in patients with paraneoplasia |
|  | 1. Metabolic disorders |
|  | 1. Psychocutaneous diseases |

^1^ Includes malassezia folliculitis.

^2^ Includes seborrhoeic dermatitis.

^3^ Includes pityriasis alba, pityriasis versicolor, confluent and reticulated papillomatosis.

^4^ Includes keratosis pilaris.

^5^ Includes scarring (keloid, hypertrophic, atrophic, striae).

^6^ Includes melanocytic naevi.

^7^ Includes sunburn and callus.
